# Second-line FOLFOX chemotherapy for patients with advanced biliary tract cancers pretreated with cisplatin/gemcitabine: a systematic review and meta-analysis

**DOI:** 10.1016/j.esmogo.2024.100055

**Published:** 2024-04-23

**Authors:** A. Digklia, D. Arnold, I.A. Voutsadakis

**Affiliations:** 1 Department of Oncology, Lausanne University Hospital, Lausanne, Switzerland; 2 Department of Oncology and Hematology, Asklepios Tumorzentrum Hamburg, AK Altona, Hamburg, Germany; 3 Algoma District Cancer Program, Sault Area Hospital, Sault Ste Marie; 4 Division of Clinical Sciences, Section of Internal Medicine, Northern Ontario School of Medicine, Sudbury, Canada

**Keywords:** biliary cancer, metastatic, FOLFOX, meta-analysis, second-line treatment

## Abstract

**Background:**

Biliary cancers are aggressive carcinomas frequently diagnosed at an advanced stage. Palliative combination systemic therapy provides survival benefits in the first-line setting of advanced and metastatic disease. FOLFOX chemotherapy is one of the few options in the second-line therapy.

**Materials and methods:**

The medical literature was searched through the Medline/PubMed and Embase databases to acquire clinical reports or trials of FOLFOX treatment for biliary cancers in the second-line metastatic setting after first-line cisplatin/gemcitabine chemotherapy. Eligible prospective and retrospective studies were reviewed and included in a meta-analysis with overall response rate (ORR), disease control rate (DCR), progression-free survival (PFS), and overall survival (OS) as outcomes of interest.

**Results:**

Six clinical studies were eligible and included in the meta-analysis. The ORR with second-line FOLFOX chemotherapy in this population was 10.42% [95% confidence interval (CI) 4.55% to 16.3%]. Two-fifths of the patients had stable disease for a DCR of 50.65% (95% CI 38.4% to 62.9%). The median PFS was 3.03 months (95% CI 1.38-4.09 months) and the median OS was 6.43 months (95% CI 5.43-7.43 months). The main grade 3/4 adverse effects observed in >10% of patients were neutropenia (21.2%) and asthenia/fatigue (10.3%).

**Conclusions:**

The meta-analysis observed a moderate efficacy of the FOLFOX combination in this setting. These results may be used as a benchmark to compare gains obtained in this setting with novel treatments, including recently introduced targeted therapies in appropriately selected patients.

## Highlights

• Biliary tract cancers are a rare type of cancer that often present in advanced stages and remain difficult to treat, resulting in high mortality rates.
• Second-line FOLFOX appears to be an effective treatment for advanced-stage biliary tract cancers.
• FOLFOX has a manageable toxicity profile, with the most common type of adverse event being hematologic; by contrast, the incidence of nonhematologic adverse events remained relatively low.

## Introduction

Biliary tract cancers (BTCs), encompassing intrahepatic and extrahepatic cholangiocarcinomas, gallbladder carcinomas, and ampulla of Vater carcinomas, are rare malignancies with an estimated incidence, together with liver cancers, of ∼53 000 cases in the United States.^1^ Mortality from these diseases is high, with an estimated death toll of >33 000 patients in the United States in 2023.^1^ Currently, Asia has the highest incidence and mortality rates, whereas European countries have lower rates, which is consistent with the lack of well-known infection-related risk factors.^2^ BTCs represent ∼5% of all cancers in incidence and 10% of all cancer-related deaths. Locally advanced unresectable and metastatic stages comprise two-thirds of all BTCs at diagnosis and have a poor prognosis with a 5-year survival rate of <5%.^3^ Among biliary cancers, extrahepatic and ampulla of Vater carcinomas are the most common, representing 60%-70% of all cases, whereas intrahepatic and gallbladder carcinomas are less frequent, representing 20% and 10% of cases, respectively.^1^^,^^2^

The first-line treatment for metastatic BTCs relies on systemic cisplatin/gemcitabine-based chemotherapy with the recent addition of durvalumab or pembrolizumab immunotherapy.^4^, ^5^, ^6^ A triplet chemotherapy combination with FOLFIRINOX was not superior to cisplatin/gemcitabine in a randomized phase II trial.^7^ Besides, the intensification of doublet chemotherapy with the adjunction of nab-paclitaxel has not been prospectively confirmed to confer meaningful clinical benefit, compared with doublets.^7^^,^^8^

Beyond the first line, treatment of patients with advanced and metastatic BTC with chemotherapy offers only borderline benefits, and there is no clear consensus on the best regimen. Recently, trials of second-line therapy with 5-fluorouracil-based regimens have confirmed a significant, albeit small, benefit.^9^, ^10^, ^11^

Genomic studies based on next-generation sequencing have revealed molecular alterations in a significant minority of cholangiocarcinomas that could be targeted with specific drugs. Intrahepatic cholangiocarcinomas, for example, present isocitrate dehydrogenase 1 (*IDH1*) or *IDH2* mutations in ∼20%-25% of cases.^12^ In addition, ∼15% of cases possess fibroblast growth factor receptor 2 (FGFR2) alterations, 5%-10% of cases have *v-raf* murine sarcoma viral oncogene homolog B1 (*BRAF*) mutations, and 10% of cases have Kirsten rat sarcoma virus (*KRAS*) mutations. A smaller percentage of cholangiocarcinomas bear human epidermal growth factor receptor 2 (HER2) amplifications, neurotrophic tyrosine receptor kinase (NTRK) alterations, and mismatch repair defects. However, not all these molecular defects are currently targetable and have proven clinical activity in BTC. For example, *KRAS* G12C mutations that may be targeted by specific inhibitors represent only ∼1 in 10 *KRAS* mutations or <1% of all intrahepatic cholangiocarcinomas, while the rest of *KRAS* mutations remain nontargetable.^11^ Still, recent data on multi-RAS inhibitors (Revolution Medicine RMC-6236) have demonstrated interesting clinical activity on other well-represented mutations in pancreatic and lung cancer including G12D, G12V, and G12R.^13^ In another example, in 15%-20% of BTC FGFR signaling pathway is activated. Since April 2020, two FGFR tyrosine kinase inhibitors, pemigatinib and futibatinib, have been approved by the Food and Drug Administration and the European Medicines Agency for the second line and beyond for disease-bearing fusions or rearrangements involving the FGFR2 receptors, based on clinical trials showing important clinical activity with high overall response rate (ORR) and long-lasting progression-free survival (PFS). Although treatment of cholangiocarcinomas with FGFR2 fusions using FGFR inhibitors has demonstrated efficacy, targeting FGFR mutations remains less established, although several case reports suggest some activity in cases with specific mutations.^14^ Thus chemotherapy remains the only second-line option for most patients with BTCs without targetable molecular alterations.

In this systematic review and meta-analysis, we compile data from clinical trials and other published series to define the efficacy and safety of second-line chemotherapy with the FOLFOX regimen for patients with locally advanced or metastatic cholangiocarcinoma.

## Materials and methods

The Medline/PubMed and Embase databases were interrogated using the search terms ‘biliary cancer’ or ‘cholangiocarcinoma’ and ‘second line chemotherapy’. Inclusion criteria for retention of retrieved articles were studies of second-line chemotherapy treatment with the FOLFOX regimen of metastatic or locally advanced, inoperable BTCs, reported after 2008. Studies reported up to 18 July 2023 were included. Both prospective and retrospective designs were allowed. Included articles had to report data in at least one of the four efficacy outcomes of interest: ORR, disease control rate (DCR), PFS, and overall survival (OS). ORR was defined as the sum of complete response and partial response and DCR was defined as ORR plus stable disease rate. Articles in languages other than English or French were excluded. We also excluded studies reporting on chemotherapies other than FOLFOX, targeted therapies, and any therapies in the first line or beyond the second line. Studies reporting on combinations of FOLFOX with other drugs were also excluded. Case reports or case series with <20 patients were excluded as well as reports on BTCs using locoregional therapy modalities. References of articles deemed appropriate for inclusion in the review were manually scanned for additional reports of interest. The risk of bias in each included article was assessed with guidance from the Risk Of Bias In Non-randomised Studies - of Interventions (ROBINS-I) tool.^15^

The included articles were evaluated for demographic data of the patient population treated with the FOLFOX regimen. Recorded characteristics to include in the report were patient age, sex, Eastern Co-operative Oncology Group performance status, location of primary cancer (intrahepatic, extrahepatic, gallbladder, or ampulla of Vater), and metastatic or locally advanced stage. Information on the most common toxicities and their grade were also recorded.

The heterogeneity among the studies was evaluated with Cochran’s *Q* test and the *I*^2^ test. By default, if the studies included in the meta-analysis of each outcome showed a low between-study heterogeneity, the fixed effects model was used for the calculation of the effect summary. In the alternative scenario, wherein between-study heterogeneity was high for an outcome of interest, a random effects model was constructed for the calculation of the effect summary statistic.^16^ All meta-analysis calculations were carried out with Excel (Microsoft Corporation, Redmond, WA), based on a previously described method with adjustments as required.^17^ Significance calculations were carried out online at the Social Science Statistics site (socscistatistics.com).

## Results

**Figure 1:**
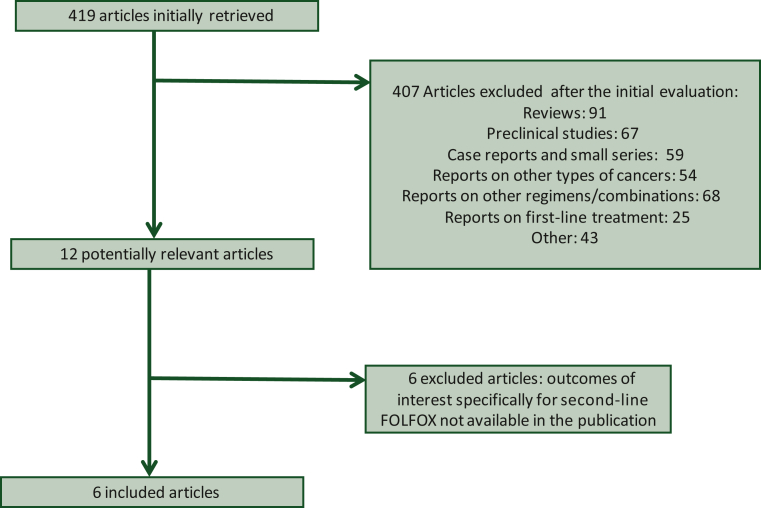
Flow diagram of articles screened for the current meta-analysis and their disposition.

The initial search of the databases retrieved 419 articles. Following a review of the titles or abstracts, we excluded 407 articles. The most common reasons for exclusion were that the articles described reviews (*n* = 91 publications), referred to preclinical studies (*n* = 67 publications), were case reports or small series with <20 patients (*n* = 59 publications), and were referring to other types of primary cancers (*n* = 54 publications) or other regimens (Figure 1). Among the remaining 12 publications, 6 were excluded because they contained patients treated with a variety of regimens in the second line, and outcomes specifically for patients treated with FOLFOX could not be extracted from the data provided.^18^, ^19^, ^20^, ^21^, ^22^, ^23^ Thus six studies published between 2014 and 2021 were finally included in the meta-analysis (Table 1).^9^^,^^24^, ^25^, ^26^, ^27^, ^28^ One of the studies was a phase III trial, three publications described phase II trials, one observational case series, and a retrospective case series. Two publications were from European countries and four were from Asia (Table 1). A total of 294 patients with biliary cancer (283 assessable for response) were described in the six studies. The risk of bias was moderate in five of the six studies included, and low in the phase III trial. The main bias was in the domain of calculation of outcomes in the observational and retrospective series, as well as selection bias in the same studies, given that patients without available data for inclusion in the series may not have had the same characteristics and outcomes as the included patients. Some outcomes of interest were not available in all six studies, potentially introducing missing data bias.

**Table 1:** The eight studies included in the meta-analysis of FOLFOX chemotherapy in the second-line treatment of patients with metastatic biliary cancer

Study author and reference	Year of publication	Type	Country	Number of patients	RR, %	DCR, %
Lamarca et al.^9^	2021	Phase III	United Kingdom	81 (80 assessable for response)	5	33.8
Choi et al.^24^	2021	Phase II	Korea	59 (51 assessable for response)	5.9	66.7
Brieau et al.^25^	2015	Retrospective	France	21	9.5	42.9
He et al.^26^	2014	Phase II	China	37	21.6	62.2
Dodagoudar et al.^27^	2016	observational	India	66	24.2	59.1
Hwang et al.^28^	2015	Phase II	Korea	30 (28 assessable for response)	7.1	46.4

DCR, disease control rate; RR, response rate.

The median ages of patients included in the six studies were between 52.5 and 65 years (range 26 and 84 years; Table 2). Slightly more patients (52.8%) were male than female (47.2%). The great majority of patients with data available (88.9%) had Eastern Co-operative Oncology Group performance status of 0 or 1 and almost all patients had received a combination of cisplatin with gemcitabine as a first-line metastatic therapy except two out of thirty patients that had received gemcitabine monotherapy in a phase two trial.^28^ Almost one-half of the patients included (49.5%) had gallbladder carcinomas followed by intrahepatic carcinomas (27.1%), extrahepatic biliary carcinomas (20.1%), and ampulla of Vater carcinomas (3.3%). Most patients (86.4%) had metastatic disease and 13.6% of patients had locally advanced disease (Table 2).

**Table 2:** Patients’ characteristics and efficacy in patients receiving second-line FOLFOX chemotherapy

Characteristics	Patients	Total patients with data, *n*	Number of series with data, *n*
Age, median (range)	52.5-65 (26-84)	273	5
**Sex**			
Male	144 (52.7)	273	5
Female	129 (47.3)		
**Eastern Co-operative Oncology Group performance status**			
0-1	241 (88.9)	272	5
>1	31 (11.1)		
**Number of prior lines of chemotherapy**			
1-2	241 (53.8)	249	5
>2	115 (46.2)		
Range	1 to >4		
**Types of prior chemotherapy**			
Cisplatin/gemcitabine	292 (99.3)	294	6
Gemcitabine	2 (0.7)		
**Location of primary**			
Intrahepatic	74 (27.1)	273	5
Extrahepatic	55 (20.1)		
Gallbladder	135(49.5)		
Ampulla of Vater	9 (3.3)		
**Extend**			
Metastatic	236 (86.4)	273	5
Locally advanced	37 (13.6)		
**Efficacy**			
OS (months), median (95% CI)	6.43 (5.43-7.43)	273	5
PFS (months), median (95% CI)	3.03 (1.98-4.09)	273	5
RR% (95% CI)	10.42 (4.55 to 16.3)	283	5
DC% (95% CI)	50.65 (38.4 to 62.9)	283	5

Data are presented as *n* (%) unless indicated otherwise. The third and fourth columns contain information on the total number of patients and the number of studies the result depicted in the second column is based on.

DCR, disease control rate; OS, overall survival; PFS, progression-free survival; RR, response rate.

**Figure 2:**
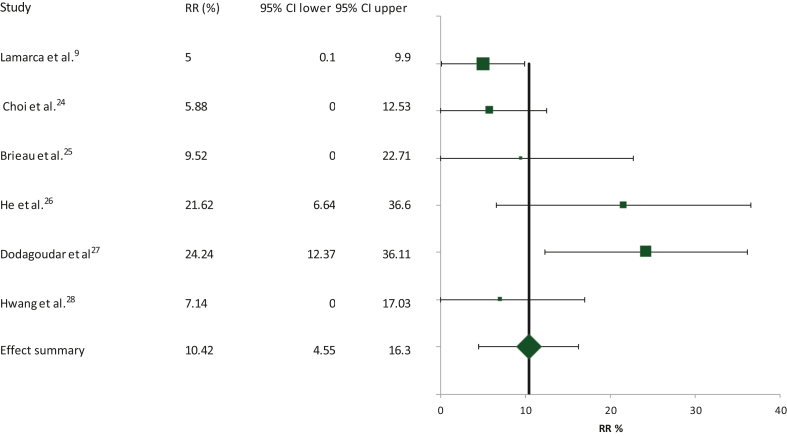
Schematic of the meta-analysis for the response rate (RR) of second-line FOLFOX in patients with advanced/metastatic biliary tract cancers who had progressed on first-line cisplatin/gemcitabine. CI, confidence interval.

Data for the calculation of response rate were available in all six studies included in the meta-analysis with 283 assessable patients. The summary response rate was 10.42% [95% confidence interval (CI) 4.55% to 16.3%; Figure 2]. Most observed responses were partial, whereas the complete response rate was 1.1%. The heterogeneity between the included studies was high with Cochran’s *Q* test equal to 12.26 (χ^2^ *P* = 0.03 for *df* = 5) and the *I*^2^ test equal to 59.23. Thus the random effects model was applied in the effect summary calculations.

**Figure 3:**
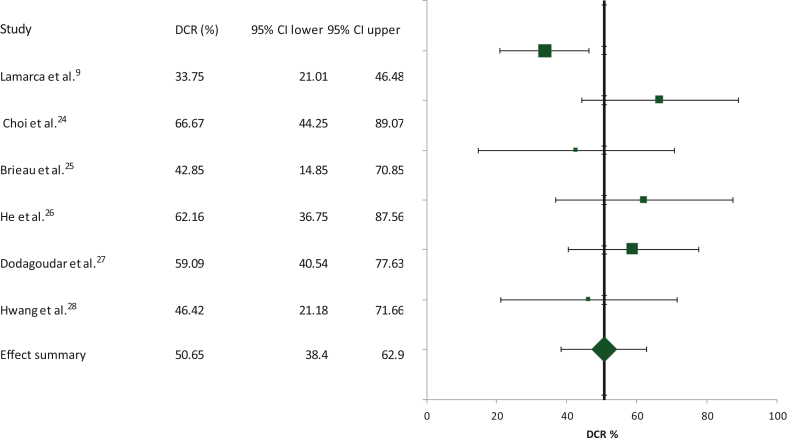
Schematic of the meta-analysis for the disease control rate (DCR) of second-line FOLFOX in patients with advanced/metastatic biliary tract cancers who had progressed on first-line cisplatin/gemcitabine. CI, confidence interval.

The calculation of DCR was based on all six studies included in the meta-analysis with 283 patients. The summary DCR was 50.65% (95% CI 38.4% to 62.9%; Figure 3). In this case, the heterogeneity between studies was moderate (Cochran’s *Q* test = 10.18, χ^2^ *P* = 0.07, *I*^2^ test = 50.9) and the random effects model was preferred.

**Figure 4:**
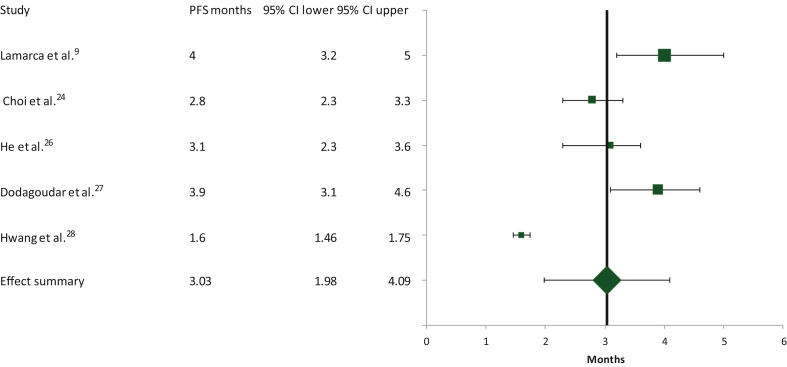
Schematic of the meta-analysis for the progression-free survival (PFS) of second-line FOLFOX in patients with advanced/metastatic biliary tract cancers who had progressed on first-line cisplatin/gemcitabine. CI, confidence interval.

Calculations of the summary effect for PFS were based on five studies with 273 assessable patients, as one of them did not provide data for the calculation of PFS.^25^ The median PFS obtained was 3.03 months (95% CI 1.98-4.09 months; Figure 4). The heterogeneity observed between the studies included was high (Cochran’s *Q* test = 90, χ^2^ *P* < 0.0001, *I*^2^ test = 90.5), prompting the use of the random effects model in this case too.

**Figure 5:**
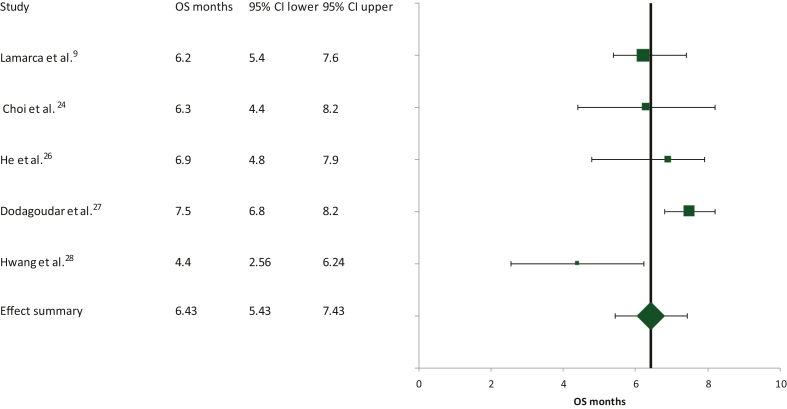
Schematic of the meta-analysis for the overall survival (OS) of second-line FOLFOX in patients with advanced/metastatic biliary tract cancers who had progressed on first-line cisplatin/gemcitabine. CI, confidence interval.

The median OS derived from the same five studies included in the PFS calculations was 6.43 months (95% CI 5.43-7.43 months; Figure 5). In these calculations, heterogeneity was high (Cochran’s *Q* test = 11.78, χ^2^ *P* = 0.01, *I*^2^ test = 66.1), requiring the use of a random effects model.

The most frequent all-grade adverse effects with second-line FOLFOX chemotherapy in patients with BTC pretreated with cisplatin/gemcitabine were asthenia/fatigue, which was observed in about two-thirds of the patients; neutropenia (56%); and nausea/vomiting, which was observed in 52.7% of the patients (Table 3). The most frequent grade 3/4 adverse effect was neutropenia (21.2%), followed by asthenia/fatigue, which was the only other grade 3/4 adverse effect observed in >10% of patients. Other grade 3/4 adverse events observed in >5% of patients were biliary events (7.8%), nausea/vomiting (7%), anemia (6.2%), thrombocytopenia (5.8%), and diarrhea (5.4%; Table 3).

**Table 3:** Toxicity of FOLFOX chemotherapy in patients with metastatic biliary cancer

Toxicity	All grades, %	Total patients with data/series with data	Grades 3 and 4, %	Total patients with data/series with data
Neutropenia	56	273/5	21.2	273/5
Anemia	37.7	273/5	6.2	243/4
Thrombocytopenia	33	273/5	5.8	243/4
Asthenia/fatigue	67.8	273/5	10.3	243/4
Anorexia	44.7	273/5	0.4	243/4
Diarrhea	30.6	147/2	5.4	147/2
Constipation	27.1	177/3	1.1	177/3
Nausea/vomiting	52.7	273/5	7	243/4
Mucositis/stomatitis	32.2	273/5	3.7	243/4
Biliary event	30.9	243/4	7.8	243/4
Peripheral neuropathy	48	273/5	1.6	243/4

The third and fifth columns contain information on the total number of patients and the number of series the percentage depicted in the second and fourth columns is based on.

## Discussion

BTCs are a rare type of cancer that often present in advanced stages and remain difficult to treat, resulting in high mortality rates. When diagnosed with localized resectable disease, patients with biliary cancers are treated with surgery, adjuvant chemotherapy, and/or radiotherapy.^29^ Nearly all patients with lymph node metastases eventually develop primary or distant relapse. In the advanced and metastatic setting, first-line therapy consists of a combination of chemotherapy and immunotherapy, based on phase III trial evidence.^5^^,^^6^

Options for later-line treatment of patients with advanced biliary tract carcinomas, after progression on first-line chemotherapy and immunotherapy, remain limited. The only approved treatment in this setting, which demonstrates a modest OS benefit compared with placebo, is the FOLFOX regimen. In the ABC-06 trial that compared FOLFOX with placebo, the median OS in the FOLFOX arm was 6.2 months versus 5.3 months in the placebo arm.^8^ The OS rates at 12 months were 25.9% and 11.4% respectively.

Liposomal irinotecan with 5-fluorouracil and leucovorin is an alternative regimen that was examined in a phase II trial of metastatic BTCs in the second-line setting and was compared with 5-fluorouracil and leucovorin.^10^ The triplet combination fared better than 5-fluorouracil and leucovorin and produced a median PFS, as assessed by investigator review, of 3.9 months (95% CI 2.7-5.2 months) and a median OS of 8.6 months (95% CI 5.4-10.5 months).^10^ Another option in the second-line setting is intensification of chemotherapy with FOLFIRINOX, which contains both oxaliplatin and irinotecan. FOLFIRINOX was studied in recent proof-of-concept clinical trials.^28^^,^^29^ In a single-arm phase II trial, a modified FOLFIRINOX schedule has demonstrated a PFS of 6.2 months and OS of 10.7 months as salvage treatment in 40 patients.^30^ Interestingly, 14 patients (46.7%) had disease control of at least 4 months. In another prospective study, 34 patients with advanced BTCs who failed first-line gemcitabine-based chemotherapy were treated with mFOLFIRINOX.^31^ The objective response rate was 14.7%. The median PFS and OS were 2.8 months (95% CI 1.6-4.0 months) and 6.2 months (95% CI 5.0-7.4 months), respectively. Dose reductions were required in 40% of patients. The toxicity profile was manageable and the most common type of adverse events were hematologic, whereas the incidence of nonhematologic adverse events was relatively low. The incidence of grade ≥3 peripheral neuropathy was 5%.

Molecular profiling is becoming increasingly important for successful therapy in several advanced cancers. As discussed earlier, several targeted treatments have been approved for BTCs with specific molecular alterations, including IDH1 mutations, ERBB2 amplifications or overexpression, canonical BRAF V600E mutations, NTRK and RET fusions, and fusions involving the *FGFR2* gene.^32^, ^33^, ^34^ In this context, next-generation sequencing testing is strongly recommended in all patients with advanced BTC to identify those that can benefit from personalized targeted treatment.^35^

Still, these alterations concern only a rather small subset of patients. In addition, the costs of these targeted drugs are prohibitive for several patients with no health benefits. As a result, most patients with advanced biliary tract carcinomas will continue to receive standard chemotherapy treatments as their best second-line option. In this context, the current report compiling data from clinical trials and other patient series on second-line FOLFOX chemotherapy provides valuable clinical information about the efficacy of this standard regimen in various settings and countries. Furthermore, FOLFOX, as a standard second-line option, will be one of the regimens to use as a reference when new treatments are introduced in these cancers.

Whether targeted therapies should be used before FOLFOX in the second-line setting in patients with molecular alterations remains a question that will require evaluation in prospective randomized trials. However, such trials may prove difficult to conduct because of the rarity of individual molecular alterations and the availability of multiple competing drugs for each alteration. Furthermore, with the establishment of checkpoint inhibitors in the first line, the efficacy of standard chemotherapies and targeted therapies as second-line therapies will have to be reassessed.

In this systematic review and meta-analysis of second-line chemotherapy for advanced or metastatic biliary tract carcinomas, six studies with 283 patients were included. The median age of the patients in the included studies ranged from 52.5 to 65 years, which is slightly younger than the typical age at diagnosis of biliary cancers in the United States.^36^ This may relate to the fact that most studies included in the meta-analysis were from Asia. However, even in the West, ∼10% of patients with biliary tract carcinoma present at a young age (<50 years).^36^

The FOLFOX regimen as a second-line treatment in patients with advanced/metastatic BTC showed an ORR of ∼10%, while about one-half of the patients achieved either a response or stability of their disease. The median PFS was ∼3 months and the median OS was just short of 6.5 months. These survival outcomes were very similar to the survival outcomes observed with the FOLFOX regimen in the prospective ABC-06 trial, which was one of the studies included in this meta-analysis. The median PFS was also similar to that observed using the liposomal irinotecan with 5-fluorouracil and leucovorin regimen in the NIFTY trial, which included patients from Asia.^10^ The liposomal irinotecan-based regimen showed a slightly better median OS of 8.6 months. The adverse effect profile of FOLFOX, as delineated in this review, was also similar to the incidence of adverse effects observed in these trials.

A limitation of this meta-analysis stems from the fact that the number of eligible studies was small and a moderate number of patients were evaluated. In addition, four of the six studies included in the meta-analysis were carried out in Asia. Thus it remains less clear whether our results accurately reflect the efficacy of the regimen in Western populations. However, as mentioned earlier, the observed outcomes were similar to those of the FOLFOX arm of the AC-06 trial, which recruited globally. Another limitation of this meta-analysis is that some studies did not provide results from certain outcomes of interest, thus decreasing the total number of patients analyzed and creating possible missing data bias. Lastly, genomic analyses were not available in the included studies, and thus no conclusions regarding the efficacy of the FOLFOX regimen in patients with specific biliary tract carcinoma alterations can be derived from the current analysis.

Advanced biliary tract carcinomas remain rare but aggressive malignancies that are increasing in incidence.^37^ Although the current literature supports the role of chemotherapy as well as the use of molecular profiling to identify alternative personalized treatment options, there remains an urgent, unmet need to develop more efficacious therapies. In this context, it is important to design clinical trials comparing the efficacy of these targeting agents with standard chemotherapy. Using the best supportive care alone as the comparator arm is not advisable. The superior efficacy reported in prospective studies, confirmed in our meta-analysis, along with an acceptable adverse effect profile, supports combination chemotherapy as the preferred standard option for patients capable of receiving treatment. In addition to survival, continuous focus on quality-of-life measurements should be part of the clinical trials evaluating novel targeted therapies.
